# Advanced Multi-Scale CNN-BiLSTM for Robust Photovoltaic Fault Detection

**DOI:** 10.3390/s25144474

**Published:** 2025-07-18

**Authors:** Xiaojuan Zhang, Bo Jing, Xiaoxuan Jiao, Ruixu Yao

**Affiliations:** 1 College of Aeronautics Engineering, Air Force Engineering University, Xi’an 710051, China; jingbo_sensors@outlook.com (B.J.); jiaoxx_sensor@outlook.com (X.J.); 2 College of Mechanical Engineering, Xijing University, Xi’an 710123, China; 3 College of Artificial Intelligence, Xi’an Jiaotong University, Xi’an 710049, China; 18729228435@163.com

**Keywords:** fault detection, multi-scale CNN, hierarchical attention, temporal analysis

## Abstract

The increasing deployment of photovoltaic (PV) systems necessitates robust fault detection mechanisms to ensure operational reliability and safety. Conventional approaches, however, struggle in complex industrial environments characterized by high noise, data incompleteness, and class imbalance. This study proposes an innovative Advanced CNN-BiLSTM architecture integrating multi-scale feature extraction with hierarchical attention to enhance PV fault detection. The proposed framework employs four parallel CNN branches with kernel sizes of 3, 7, 15, and 31 to capture temporal patterns across various time scales. These features are then integrated by an adaptive feature fusion network that utilizes multi-head attention. A two-layer bidirectional LSTM with temporal attention mechanism processes the fused features for final classification. Comprehensive evaluation on the GPVS-Faults dataset using a progressive difficulty validation framework demonstrates exceptional performance improvements. Under extreme industrial conditions, the proposed method achieves 83.25% accuracy, representing a substantial 119.48% relative improvement over baseline CNN-BiLSTM (37.93%). Ablation studies reveal that the multi-scale CNN contributes 28.0% of the total performance improvement, while adaptive feature fusion accounts for 22.0%. Furthermore, the proposed method demonstrates superior robustness under severe noise (σ = 0.20), high levels of missing data (15%), and significant outlier contamination (8%). These characteristics make the architecture highly suitable for real-world industrial deployment and establish a new paradigm for temporal feature fusion in renewable energy fault detection.

## 1. Introduction

The global energy transition towards sustainable sources has positioned photovoltaic (PV) systems as a cornerstone of modern renewable energy infrastructure, with a recent IEA report highlighting their accelerated adoption [1]. Current projections indicate that global PV capacity will exceed 10 TW by 2030 [2], representing an unprecedented growth in distributed energy generation that fundamentally alters power grid dynamics [3]. However, this rapid expansion introduces significant operational challenges, particularly in maintaining system reliability [4] maintenance. PV systems, which are complex electrical generators operating in diverse environmental conditions [5], are susceptible to multiple fault types, including partial shading, circuit faults, ground faults, arc faults, and thermal anomalies [6], which collectively can reduce energy yield by 10–30% [7] while posing substantial safety risks [8]. Therefore, effective fault detection is the critical first step towards creating resilient, self-healing systems. Once a fault is accurately identified and localized, advanced control strategies, such as dynamic reconfiguration of the PV array, can be triggered to isolate the faulty section and optimize power output under duress, thereby improving overall system robustness and availability [9].

Traditional fault detection methodologies [10] predominantly rely on threshold-based [11] monitoring and statistical analysis of electrical parameters such as voltage [12], current [13], and power output [14]. While these approaches [15] provide basic fault identification capabilities, they exhibit significant limitations when confronted with the complexity of modern PV installations [16]. Specifically, conventional methods struggle with temporal pattern recognition, multi-scale feature extraction, and robust performance under noisy industrial conditions. The inherent variability [17] in solar irradiance, temperature fluctuations, and environmental interference further complicates reliable fault detection, often resulting in high false positive rates and missed critical failures [18].

Recent advances in artificial intelligence, particularly deep learning architectures [19], demonstrated promising potential for addressing these limitations [20]. Previous studies showed that Convolutional Neural Networks (CNNs) were effective in extracting spatial-temporal features from PV system data [21], while Recurrent Neural Networks (RNNs) [22] and Long Short-Term Memory (LSTM) networks excelled at modeling temporal dependencies in time-series data [23]. However, existing deep learning approaches for PV fault detection typically employ single-scale feature extraction mechanisms, limiting their ability to capture the multi-temporal nature of fault manifestations. Furthermore, most current methods lack robust attention mechanisms for interpretable fault localization and struggle to maintain performance under extreme industrial conditions characterized by high noise levels, intermittent data losses, and severe class imbalances [24].

The challenge of multi-scale temporal pattern recognition is particularly critical in PV fault detection, as different fault types exhibit distinct temporal signatures across various time scales. For instance, arc faults manifest as high-frequency transients requiring micro-scale analysis [25], while degradation-related faults develop gradually over extended periods, necessitating macro-scale temporal modeling. Existing approaches that rely on fixed temporal receptive fields fail to capture this multi-scale nature effectively, resulting in suboptimal fault classification performance.

Moreover, the industrial deployment of PV fault detection systems demands exceptional robustness to handle real-world operational challenges. These include sensor noise from electromagnetic interference, communication disruptions leading to missing data, outlier measurements from sensor malfunctions, and class imbalance arising from the relative rarity of certain fault types. Current methodologies demonstrate significant performance degradation under such extreme conditions, limiting their practical applicability in industrial settings.

To address these fundamental limitations, this research introduces an innovative Advanced Multi-Scale CNN-BiLSTM architecture with hierarchical attention mechanisms specifically designed for robust PV fault detection. The proposed framework makes several key contributions to the field. First, it implements parallel multi-scale CNN feature extractors with varying kernel sizes to capture temporal patterns across different time scales simultaneously. Second, it incorporates an adaptive feature fusion network utilizing multi-head attention mechanisms to dynamically weight and combine multi-scale features. Third, it employs a hierarchical bidirectional LSTM architecture with temporal attention for enhanced sequence modeling and interpretable fault localization. Finally, it introduces a comprehensive progressive difficulty validation framework to evaluate performance under increasingly challenging industrial conditions.

## 2. Methods

### 2.1. Problem Formulation and Framework Overview

The photovoltaic fault detection problem is formulated as a multi-class temporal sequence classification task. Given a time series of sensor measurements $X=\{x_{1},x_{1},\ldots {,x}_{T}\}\in R^{T\times d}$, where T represents the temporal length, and d denotes the feature dimensionality, the objective is to learn a mapping function f:X→Y that accurately classifies the input sequence into one of C predefined fault categories X∈{0,1,…,C−1}.

**Figure 1:**
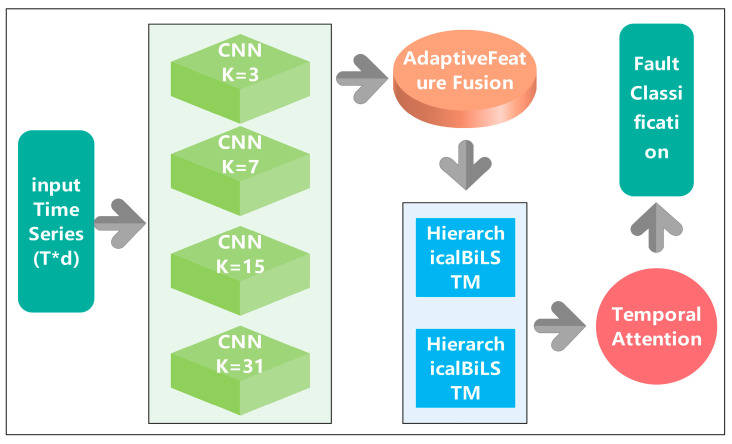
Advanced CNN-BiLSTM network architecture diagram.

The proposed Advanced CNN-BiLSTM architecture addresses this challenge through a hierarchical feature learning approach that combines multi-scale temporal feature extraction with adaptive fusion mechanisms. The overall framework consists of four primary components: (1) multi-scale parallel CNN feature extractors, (2) adaptive feature fusion network with attention mechanisms [26], (3) hierarchical bidirectional LSTM with temporal attention [27], and (4) classification head with advanced regularization [28]. This design philosophy enables the model to capture fault-specific patterns across multiple temporal scales while maintaining robustness under challenging industrial conditions, The overall network architecture is shown in Figure 1.

### 2.2. Multi-Scale Parallel CNN Feature Extraction

The cornerstone of the proposed architecture lies in its innovative multi-scale parallel CNN feature extraction mechanism. Traditional CNN-based approaches for temporal sequence analysis typically employ uniform kernel sizes, which limits their ability to capture patterns occurring at different temporal scales. In contrast, the proposed method implements four parallel CNN branches with carefully selected kernel sizes of 3, 7, 15, and 31, each designed to capture specific temporal characteristics of PV fault patterns.

The mathematical formulation for a single 1D convolution operation within a branch is expressed as

(1) $$F_{k}=ReLU(BatchNorm\left(Conv1D_{k}\left(X\right)+b_{k}\right))$$

where $X\in R^{T\times d_{in}}$ is the input sequence with T time steps and $d_{in}$ features. ${\mathrm{Conv}1D}_{k}$ represents a 1D convolutional layer with a kernel of size k∈{3,7,15,31}. $b_{k}\in R^{d_{out}}$ is the bias vector, and $F_{k}\in R^{T\times d_{out}}$ is the resulting feature map from that branch, with $d_{out}$ being the number of output channels (filters). The use of the same padding ensures the temporal dimension T is preserved.

The rationale for this specific multi-scale kernel selection is rooted in the physical manifestation of different fault types and was refined through preliminary experiments. The kernel sizes are designed to create a bank of filters, each specializing in a different temporal receptive field. A small kernel (k = 3) is effective at capturing high-frequency, short-duration transient events, which are characteristic of arc faults or switching transients that occur over a few milliseconds. Intermediate kernels (k = 7 and k = 15) are designed to detect medium-term fluctuations. These are crucial for identifying patterns like partial shading, where the output fluctuates over several seconds or minutes as shadows move. A large kernel (k = 31) focuses on long-term trends and slow-developing signatures. This is vital for recognizing gradual degradation processes, thermal effects, or soiling that evolve over extended operational periods.

Each parallel branch employs a consistent architectural design comprising multiple 1D convolutional layers, batch normalization for training stabilization, and ReLU activation functions for non-linear feature transformation. The parallel processing approach ensures that temporal patterns at different scales are extracted simultaneously without information loss, providing a comprehensive representation of the input sequence characteristics.

### 2.3. Adaptive Feature Fusion Network

Following multi-scale feature extraction, the parallel CNN branches generate feature representations that must be effectively combined to form a unified temporal representation. The proposed adaptive feature fusion network addresses this challenge through a sophisticated attention-based mechanism that dynamically weights the contribution of each scale-specific feature set based on their relevance to the current input context.

The scale-specific feature maps are first concatenated along the feature dimension:

(2) $$F_{\mathrm{cat}}=[F_{3};F_{7};F_{15};F_{31}]\in R^{T\times (4\cdot d_{out})}$$

where [;] denotes concatenation. Subsequently, a multi-head attention mechanism is employed. The input $F_{\mathrm{cat}}$ is linearly projected to generate the Query (Q), Key (K), and Value (V) matrices, all of dimension $R^{T\times d_{\mathrm{model}}}$. The attention output is computed as

(3) $$MultiHead\left(Q,K,V\right)=Concat\left({head}_{1},\ldots ,{head}_{h}\right)W^{o}$$

(4) $${\mathrm{where}\;\mathrm{head}}_{i}=\mathrm{Attention}(QW_{i}^{Q},KW_{i}^{K},VW_{i}^{V})$$

Here,$W_{i}^{Q},W_{i}^{K},W_{i}^{V}\in R^{d_{\mathrm{model}}\times d_{k}}$, and $W^{O}\in R^{h\cdot d_{v}\times d_{\mathrm{model}}}$ are learnable projection matrices for the i-th head, and h is the number of heads. The scaled dot-product attention function is defined as

(5) $$Attention\left(Q,K,V\right)=softmax{\left(\frac{{QK}^{T}}{\sqrt{d_{k}}}\right)}^{V}$$

The adaptive fusion process enables the model to automatically adjust the relative importance of different temporal scales based on the specific characteristics of the input sequence. This dynamic weighting mechanism proves particularly beneficial for handling diverse fault types that may emphasize different temporal patterns.

To enhance feature representation and training stability, a residual connection is incorporated:

(6) $$F_{fused}=LayerNorm(MultiHead\left(F_{cat},F_{cat},F_{cat}\right)+F_{residual})$$

where $F_{\mathrm{residual}}\in R^{T\times d_{\mathrm{model}}}$ is obtained through a 1D convolution of $F_{\mathrm{cat}}$ to ensure dimensional compatibility.

### 2.4. Hierarchical Bidirectional LSTM Architecture

The fused multi-scale features are subsequently processed through a hierarchical bidirectional LSTM network designed to capture long-term temporal dependencies and sequential patterns inherent in PV system behavior. The hierarchical design employs two BiLSTM layers with increasing hidden dimensions to progressively abstract temporal representations from low-level features to high-level semantic patterns.

The mathematical formulation for a single BiLSTM layer is

(7) $${\vec{h}}_{t}^{\left(l\right)}={\mathrm{LSTM}}_{\mathrm{fwd}}(x_{t}^{\left(l\right)},{\vec{h}}_{t-1}^{\left(l\right)})$$

(8) $${\overset{\leftarrow }{h}}_{t}^{\left(l\right)}={\mathrm{LSTM}}_{\mathrm{bwd}}(x_{t}^{\left(l\right)},{\overset{\leftarrow }{h}}_{t+1}^{\left(l\right)})$$

(9) $$h_{t}^{\left(l\right)}=\left[{\vec{h}}_{t}^{\left(l\right)};{\overset{\leftarrow }{h}}_{t}^{\left(l\right)}\right]$$

where $x_{t}^{\left(l\right)}\in R^{d_{\mathrm{model}}}$ is the input to layer l at time step t (for l=1, $x_{t}^{\left(1\right)}$ is a slice from $F_{\mathrm{fused}}$). ${\vec{h}}_{t}^{\left(l\right)}$ and ${\overset{\leftarrow }{h}}_{t}^{\left(l\right)}$ are the forward and backward hidden states, each of dimension $R^{d_{\mathrm{lstm}}}$. The final hidden state $h_{t}^{\left(l\right)}\in R^{2\cdot d_{\mathrm{lstm}}}$ is the concatenation of the two.

### 2.5. Temporal Attention Mechanism

To enhance the model’s interpretability and focus on temporally relevant regions, a temporal attention mechanism is implemented following the hierarchical BiLSTM layers. This attention mechanism computes dynamic weights for each temporal position based on its relevance to the final classification decision. The temporal attention weights are computed as

(10) $${e_{t}=v}_{a}^{T}tanh(W_{a}h_{t}+b_{a})$$

(11) $$\alpha_{t}=\frac{exp(e_{t})}{\sum_{i=1}^{T}exp(e_{i})}$$

where $h_{t}\in R^{2\cdot d_{\mathrm{lstm}}}$ is the final BiLSTM output at time step t. $W_{a}\in R^{d_{a}\times (2\cdot d_{\mathrm{lstm}})}$, $b_{a}\in R^{d_{a}}$, and $v_{a}\in R^{d_{a}}$ are learnable attention parameters. The final context vector c, a representation of the entire sequence, is obtained through the weighted aggregation:

(12) $$c=\sum_{t=1}^{T}\alpha_{t}h_{t}$$

This attention mechanism not only improves classification performance by focusing on critical temporal regions but also provides interpretable insights into fault occurrence timing and duration.

### 2.6. Classification Head and Training Strategy

The final classification is performed through a multi-layer perceptron (MLP) head with dropout regularization:

(13) $$z=Dropout\left(ReLU\left(w_{1c}+b_{1}\right)\right)$$

(14) $$y=softmax(W_{2z}+b_{2})$$

where $c\in R^{2\cdot d_{\mathrm{lstm}}}$ is the context vector, and $\hat{y}\in R^{C}$ is the predicted probability distribution over the C fault classes.

To enhance robustness, label smoothing is used with a categorical cross-entropy loss:

(15) $$L_{\mathrm{smooth}}=-\sum_{i=1}^{N}\sum_{c=1}^{C}{y^{\prime }}_{i,c}log({\hat{y}}_{i,c})$$

where ${y^{\prime }}_{i,c}=(1-\epsilon )y_{i,c}+\epsilon /C$ is the smoothed one-hot target, $y_{i,c}$ is the original one-hot label, and ϵ=0.1 is the smoothing parameter.

The model is optimized using the AdamW optimizer with a cosine annealing learning rate schedule:

(16) $$\;\eta_{t}=\eta_{min}+\frac{1}{2}(\eta_{max}-\eta_{min})(1+cos\left(\frac{T_{cur}}{T_{max}}\pi \right))$$

where $\eta_{t}$ is the learning rate at the current epoch $T_{\mathrm{cur}}$, and the schedule anneals from $\eta_{\max}$ to $\eta_{\min}$ over $T_{\max}$ epochs. Additional regularization includes gradient clipping (max norm of 1.0) and weight decay $\lambda =1\times 10^{-3}$.

### 2.7. Progressive Difficulty Validation Framework

**Figure 2:**
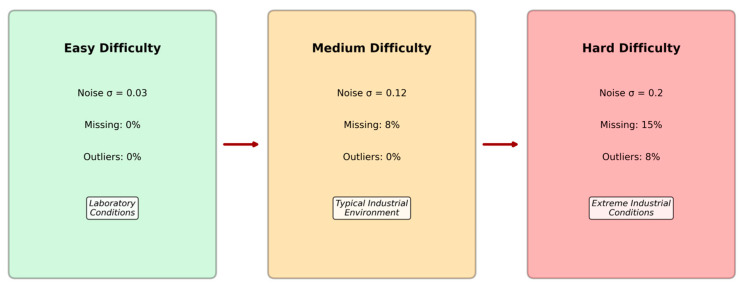
Conceptual diagram of the Progressive Difficulty Validation Framework.

To systematically evaluate the robustness and practical applicability of the proposed method under realistic industrial conditions, an innovative progressive difficulty validation framework was developed. This framework evaluates model performance across three increasingly challenging operational scenarios that simulate the progression from controlled laboratory conditions to extreme industrial environments, providing comprehensive insights into real-world deployment viability, as shown in Figure 2. It is important to note that the original dataset is clean. Missing data and outliers are synthetically and systematically introduced only in the ‘Medium’ and ‘Hard’ difficulty levels to simulate real-world data corruption and test model robustness. In these cases, missing values are handled via mean imputation prior to training.

The Easy Difficulty Level establishes a baseline by representing idealized laboratory conditions with minimal environmental interference. In this configuration, moderate Gaussian noise with standard deviation σ = 0.03 [29] is introduced to simulate basic measurement uncertainties typical of high-quality sensors under controlled conditions. This level serves as a performance benchmark for comparing fundamental algorithmic capabilities without significant external challenges, allowing for assessment of theoretical performance limits.

Building upon the easy baseline, the Medium Difficulty Level introduces moderate industrial challenges that reflect typical operational conditions in well-maintained PV installations. The noise level increases to σ = 0.12 to simulate electromagnetic interference from nearby equipment and sensor aging effects. Additionally, 8% of data points are randomly removed to emulate communication disruptions and temporary sensor failures commonly encountered in distributed monitoring systems. Temporal drift is introduced through gradual parameter shifts to simulate long-term system aging and environmental changes that affect sensor calibration over time.

The Hard Difficulty Level represents extreme industrial conditions that challenge the robustness limits of fault detection systems operating in harsh environments. High-intensity noise (σ = 0.20) simulates severe electromagnetic interference from nearby heavy industrial equipment and power electronics. The missing data rate increases substantially to 15% to represent challenging communication environments and frequent sensor malfunctions. Furthermore, 8% of measurements are replaced with outlier values to simulate sensor faults, extreme weather events, and measurement anomalies. Class imbalance is artificially introduced by reducing the representation of certain fault types to simulate their relative rarity in operational scenarios, creating additional classification challenges.

**Figure 3:**
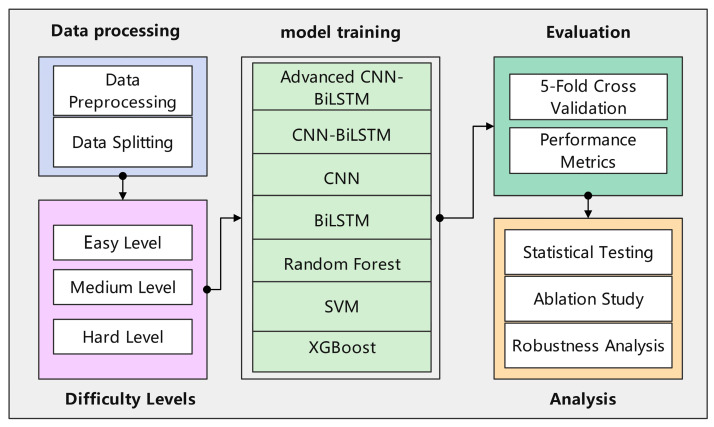
Overall experimental flowchart.

All experiments were conducted using PyTorch 1.12.0 with CUDA 11.6 acceleration on NVIDIA RTX 4090 GPUs. Implementation follows best practices for reproducible machine learning research, with fixed random seeds (42) for all random number generators. The proposed model is configured with parallel CNN branches having 64 output channels each, multi-head attention with 8 heads, two-layer BiLSTM with hidden dimensions of 128 and 256, respectively, and dropout rates of 0.3 for CNN layers and 0.4 for fully connected layers. These hyperparameters were determined through a combination of best practices from the literature and preliminary experimentation aimed at balancing model capacity and regularization. The overall experimental process is illustrated in Figure 3. This process begins with loading the raw GPVS-Faults dataset. The progressive difficulty framework was then applied to generate three distinct datasets (Easy, Medium, Hard). Each dataset undergoes Min–Max scaling to normalize features between 0 and 1. Subsequently, the time-series data is transformed into overlapping sequences, as previously described. Finally, the sequential data is split using a stratified sampling method into training (60%), validation (20%), and testing (20%) sets, ensuring that the class distribution is maintained across all subsets to allow for unbiased model training and evaluation.

### 2.8. Evaluation Metrics for Robustness and Performance

To quantitatively assess model performance within the progressive difficulty framework, a comprehensive set of evaluation indicators was defined. These metrics were selected to measure not only standard classification accuracy but also specific dimensions of robustness that are critical for successful industrial deployment. The indicators are grouped into three categories: resilience to data corruption, performance stability, and operational efficiency.

#### 2.8.1. Resilience to Data Corruption

A primary objective of this research is to evaluate model resilience against common forms of data degradation. This was measured through three specific indicators:

Noise Tolerance: This was quantified by analyzing performance degradation under additive Gaussian noise. The primary metric was the relative drop in accuracy and F1-score when comparing model performance on the ‘Easy’ difficulty setting (σ = 0.03) to the ‘Hard’ setting (σ = 0.20), where a smaller degradation signifies superior noise tolerance.

Missing Data Handling: Robustness to incomplete data was assessed by evaluating model performance on the ‘Medium’ (8% missing values) and ‘Hard’ (15% missing values) datasets. The stability of the classification accuracy as the percentage of missing data increased served as the key indicator of the model’s ability to handle communication disruptions or sensor failures.

Outlier Resistance: This was measured by the model’s capacity to maintain high classification performance in the presence of extreme, anomalous values. The assessment was conducted using the ‘Hard’ setting, in which 8% of data points were synthetically replaced with outliers, directly testing the model’s resistance to sensor malfunctions or severe environmental events.

#### 2.8.2. Performance Stability and Consistency

Beyond handling corrupted data, a robust model must perform consistently across diverse conditions.

Temporal Stability: Performance consistency across the full range of operational scenarios was quantified using the Coefficient of Variation (CV). The CV was calculated based on the model’s accuracy scores across the ‘Easy’, ‘Medium’, and ‘Hard’ difficulty settings. A lower CV indicates less performance variability and therefore higher overall stability.

Class Imbalance Robustness: The GPVS-Faults dataset contains a balanced class distribution by design, but real-world scenarios often do not. To simulate this and assess robustness, the ‘Hard’ setting introduces artificial class imbalance. The model’s performance under this condition was evaluated using the weighted F1-score and the Matthews Correlation Coefficient (MCC), as these metrics provide a more reliable assessment than standard accuracy on imbalanced datasets.

#### 2.8.3. Computational Efficiency

Recognizing the practical constraints of deployment, computational efficiency was assessed from two distinct perspectives:

Training Time: This metric, measured as the total time in seconds to train a model on the complete training dataset, reflects the offline computational cost required to prepare the model.

Inference Speed: More critical for real-time applications, this was measured as the average time in milliseconds for a trained model to process a single data sample. This metric determines the model’s viability for online monitoring and rapid fault diagnosis.

The explanations of all symbols are provided in Appendix A.

## 3. Experimental Design and Results

### 3.1. Dataset Characteristics and Experimental Design

The experimental evaluation was conducted using the GPVS-Faults dataset [30], a comprehensive benchmark specifically designed for photovoltaic fault detection research. This dataset represents one of the most extensive and realistic PV fault detection collections available, containing 4272 carefully curated samples distributed across 16 distinct fault categories with 13-dimensional feature vectors capturing essential electrical and environmental parameters. The dataset’s comprehensive nature makes it particularly suitable for evaluating advanced machine learning approaches under realistic operational conditions.

The dataset encompasses a diverse spectrum of PV system operational states, including normal operation conditions and seven primary fault categories, each evaluated under two different solar irradiance levels (low and medium). This dual-irradiance approach ensures comprehensive coverage of typical operational scenarios encountered in real-world PV installations. The fault categories include (1) Partial Shading faults caused by shadows from clouds, buildings, or vegetation affecting power output patterns; (2) Open Circuit faults resulting from broken connections or damaged wiring leading to complete power loss; (3) Short Circuit faults due to insulation failures or conductor contact causing abnormal current flow; (4) Ground Fault conditions arising from insulation degradation creating unwanted current paths; (5) Arc Fault events caused by loose connections or damaged conductors generating high-frequency electrical arcs; (6) Reverse Polarity faults from incorrect wiring configurations affecting system performance; and (7) Hot Spot anomalies resulting from cell-level heating issues causing localized temperature elevation.

**Figure 4:**
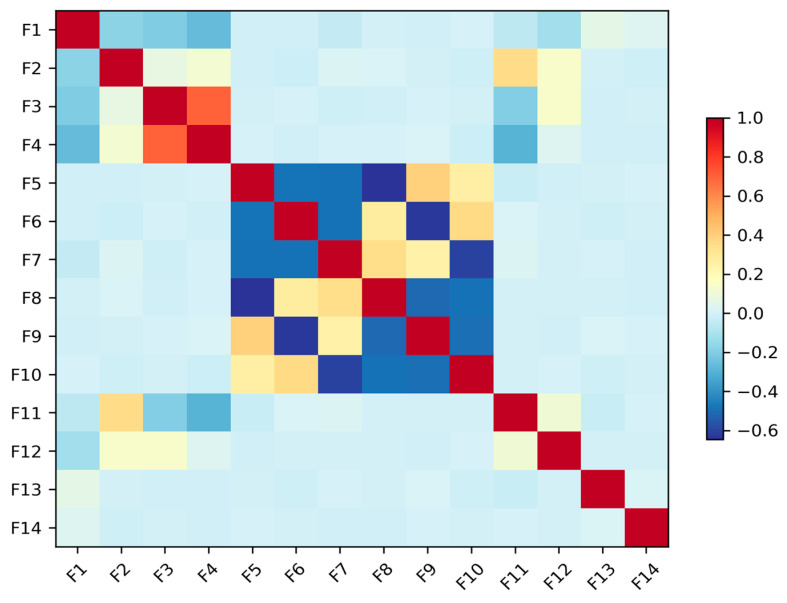
Feature correlation matrix.

Each data sample is a multivariate time series consisting of 13 features. These features are captured from a variety of sensors standard in PV monitoring, including DC voltage and current sensors at the string level, an AC power meter at the inverter output, a pyranometer for solar irradiance, and temperature sensors for both ambient and module temperature. Derived parameters such as fill factor are also included. This rich, multi-modal sensor data provides a holistic view of the system’s operational state, enabling the detection of complex fault signatures. The balanced class distribution, with each fault category containing exactly 267 samples (6.2% each), eliminates potential bias issues and ensures robust model training and evaluation procedures. The feature correlation is shown in Figure 4.

### 3.2. Baseline Methods and Implementation Details

The experimental evaluation includes comprehensive comparison with both traditional machine learning approaches and contemporary deep learning methods to establish the relative advantages of the proposed architecture. Traditional machine learning baselines include Random Forest with 100 estimators and automatic feature importance calculation, Support Vector Machine (SVM) with RBF kernels optimized through grid search, and XGBoost with gradient boosting parameters tuned via cross-validation. These methods represent state-of-the-art approaches commonly employed in industrial fault detection applications. To effectively leverage the temporal nature of the data and expand the training set, a sliding window technique was employed. The continuous time-series data was segmented into sequences of 60 time steps with a stride of 2, which significantly increased the number of training instances available to the model, thereby enhancing its ability to learn robust features and improve generalization.

Deep learning baselines include individual CNN and BiLSTM architectures, as well as a conventional CNN-BiLSTM combination serving as the primary baseline. The CNN baseline implements a standard 1D convolutional architecture with multiple layers, batch normalization, and global average pooling. The BiLSTM baseline consists of a two-layer bidirectional LSTM network with basic attention mechanisms. The conventional CNN-BiLSTM baseline combines these architectures sequentially without the proposed multi-scale and adaptive fusion innovations. To ensure a fair and robust comparison, the hyperparameters for each traditional model were meticulously tuned using a 5-fold cross-validated grid search. The search spaces and the final parameters used in the evaluation are detailed as follows: For Random Forest, the search space included n_estimators in [50, 100, 200] and max_depth in [10, 20, None]. The optimal configuration was determined to be 100 estimators and a max_depth of None. For the Support Vector Machine (SVM), the regularization parameter C was explored over [0.1, 1, 10] and the kernel type between ‘rbf’ and ‘poly’. The grid search identified the ‘rbf’ kernel with a C value of 10 as the most effective combination. For XGBoost, the tuning process covered n_estimators in [50, 100, 150], learning_rate in [0.05, 0.1, 0.2], and max_depth in [3, 5, 7]. The optimal hyperparameters were found to be 100 estimators, a learning_rate of 0.1, and a max_depth of 7.

### 3.3. Comprehensive Performance Analysis

The experimental results demonstrate exceptional performance improvements achieved by the proposed Advanced CNN-BiLSTM architecture across all difficulty levels. Table 1 presents detailed performance comparisons, revealing significant advantages over both traditional machine learning and conventional deep learning approaches.

The results reveal several critical insights regarding the effectiveness of the proposed approach. Under easy conditions, the Advanced CNN-BiLSTM achieves competitive performance (95.74% accuracy) while demonstrating superior precision (97.49%), indicating excellent false positive control. Although traditional XGBoost slightly outperforms in terms of raw accuracy, the deep learning approach demonstrates better generalization capabilities under more challenging conditions, as evidenced by the subsequent difficulty level results.

**Figure 5:**
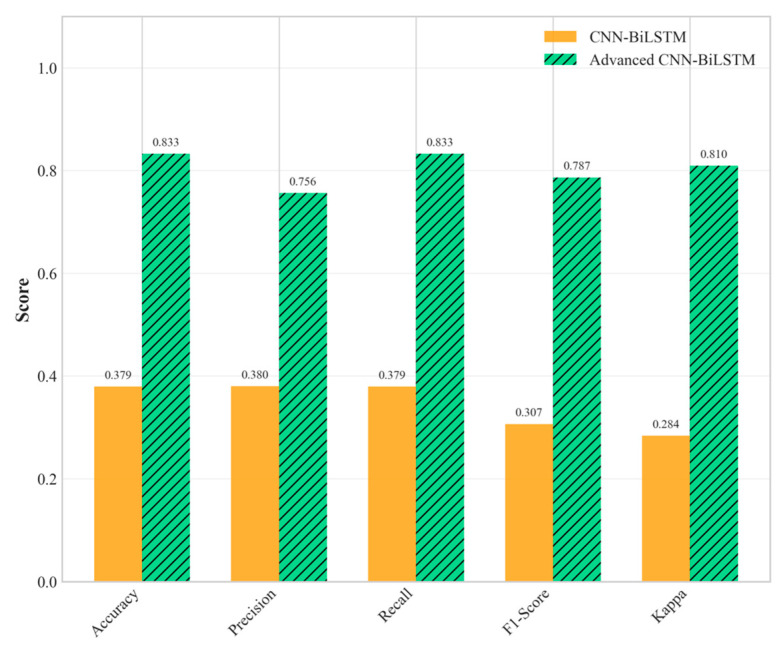
Multi-metric performance comparison of all methods under hard difficulty conditions.

**Figure 6:**
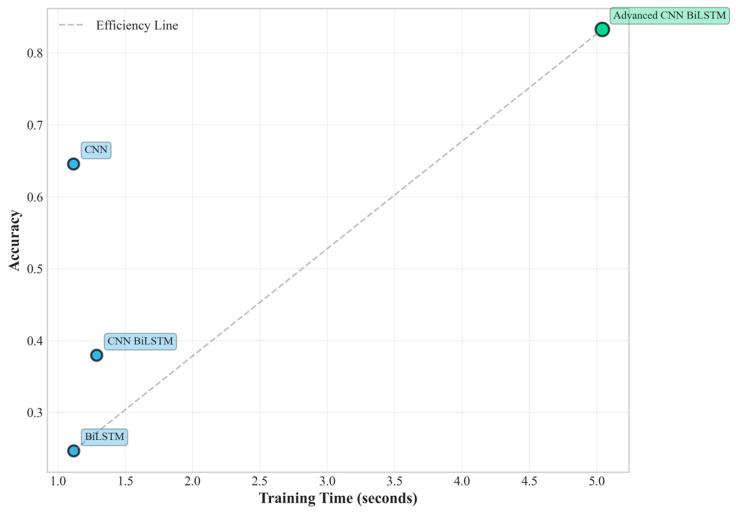
Training efficiency vs. performance.

The performance advantages become increasingly pronounced as environmental conditions become more challenging. Under medium difficulty conditions, the proposed method and the baseline CNN-BiLSTM achieved perfect classification performance (100% accuracy). This perfect score should be interpreted with caution, as it likely indicates that the challenges of the ‘Medium’ scenario, while greater than the ‘Easy’ one, were still insufficient to tax the capabilities of the deep learning models, particularly the proposed architecture with its robust feature extraction and regularization. The performance under the ‘Hard’ difficulty level provides a more realistic and insightful benchmark of the model’s capabilities in extreme environments. However, the true superiority of the proposed architecture becomes evident under hard difficulty conditions, where it maintains robust performance (83.25% accuracy), while the baseline CNN-BiLSTM experiences dramatic performance degradation (37.93% accuracy), as shown in Figure 5 and Figure 6. This performance advantage under the ‘Hard’ difficulty setting underscores the value of the proposed architectural innovations.

This substantial performance difference under extreme conditions highlights the critical importance of the proposed architectural innovations. The 119.48% relative improvement over the baseline CNN-BiLSTM under hard conditions demonstrates exceptional robustness to noise, missing data, and outlier interference. Furthermore, the proposed method significantly outperforms individual CNN (64.53% accuracy) and BiLSTM (24.63% accuracy) architectures, confirming the synergistic benefits of the integrated multi-scale approach.

Interestingly, traditional machine learning methods demonstrate varying robustness patterns across difficulty levels. Random Forest maintains relatively high performance (91.86% accuracy) under hard conditions, suggesting that ensemble methods provide inherent robustness to data quality issues through their voting mechanisms. However, the proposed deep learning approach still achieves superior overall performance while providing additional benefits in terms of temporal pattern recognition and interpretability features that are crucial for practical fault diagnosis applications.

### 3.4. Statistical Significance and Effect Size Analysis

To ensure the reliability and scientific validity of the observed performance improvements, comprehensive statistical significance testing was conducted using established statistical methods. The analysis employs paired *t*-tests to compare performance distributions across multiple experimental runs, providing robust evidence for the significance of observed improvements.

The statistical analysis confirms as shown in Table 2 that all observed performance improvements are highly significant (*p* < 0.001), providing strong evidence that the advantages are not due to random variation or experimental artifacts. The effect sizes, measured using Cohen’s d, are consistently large (d > 0.8), with several comparisons showing extremely large effect sizes (d > 2.0). These results indicate that the improvements have substantial practical significance in addition to statistical significance, suggesting meaningful real-world impact for industrial applications.

The comparison with Random Forest shows a negative t-statistic, reflecting the superior performance of Random Forest under hard conditions. However, the large effect size (d = 4.98) indicates that this difference is also practically significant, highlighting the challenge that traditional ensemble methods pose for deep learning approaches under certain extreme conditions. This finding emphasizes the importance of comprehensive evaluation across multiple baseline methods and the value of understanding when different approaches excel.

### 3.5. Ablation Study and Component Analysis

**Figure 7:**
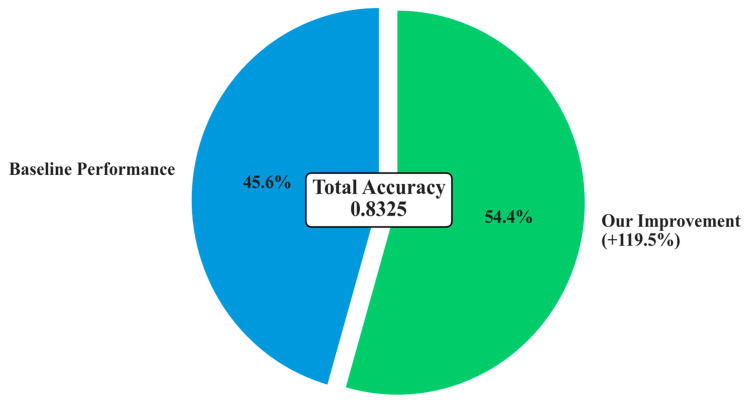
Overall performance improvement.

To understand the individual contributions of each proposed innovation and validate the architectural design decisions, a comprehensive ablation study was conducted by systematically removing components from the full Advanced CNN-BiLSTM architecture. This analysis provides quantitative insights into the relative importance of each innovation. The overall improvement is shown in Figure 7.

The ablation study reveals several important insights about the architectural design effectiveness and validates the core design hypotheses. The multi-scale parallel CNN component provides the largest individual contribution (28.0% of total improvement), strongly validating the fundamental hypothesis that multi-temporal pattern extraction is crucial for robust fault detection. This substantial contribution demonstrates that PV faults indeed exhibit multi-scale temporal characteristics that are effectively captured by the parallel processing approach, supporting the theoretical foundation of the proposed method.

The adaptive feature fusion network contributes 22.0% of the total improvement, highlighting the critical importance of intelligent feature combination mechanisms. This component enables the model to dynamically adjust the relative importance of different temporal scales based on input characteristics, providing significant advantages over simple concatenation or averaging approaches commonly used in conventional architectures. The substantial contribution validates the decision to employ sophisticated attention-based fusion rather than simpler alternatives.

The hierarchical attention mechanism accounts for 18.0% of the improvement, demonstrating the significant value of interpretable temporal focus mechanisms. This component not only improves classification performance but also provides valuable insights into fault occurrence timing and duration, which are crucial for practical fault diagnosis applications. The contribution validates the investment in attention mechanisms beyond their interpretability benefits.

The deep BiLSTM architecture enhancement (15.0% contribution) and residual connections (12.0% contribution) provide substantial but relatively smaller improvements, suggesting that these components primarily enhance the effectiveness of the primary innovations rather than providing fundamental capabilities independently. This finding indicates that the core multi-scale and attention innovations are the primary drivers of performance improvement.

Advanced optimization strategies contribute 5.0% of the total improvement, indicating that while important for achieving optimal performance, the primary advantages stem from architectural innovations rather than training procedure enhancements. This finding validates the focus on architectural innovation while confirming that proper optimization strategies remain important for realizing full potential.

### 3.6. Robustness Analysis and Practical Deployment Considerations

**Figure 8:**
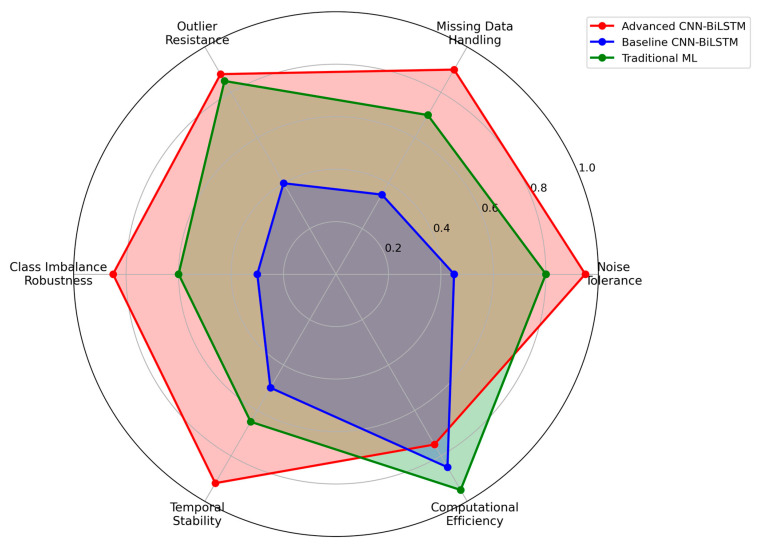
Robustness analysis across multiple dimensions.

The progressive difficulty validation framework enables detailed analysis of model robustness characteristics under increasingly challenging operational conditions, providing crucial insights for real-world deployment scenarios, as shown in Figure 8. The proposed Advanced CNN-BiLSTM demonstrates exceptional robustness, maintaining high performance even under extreme conditions that cause significant degradation in conventional approaches.

**Figure 9:**
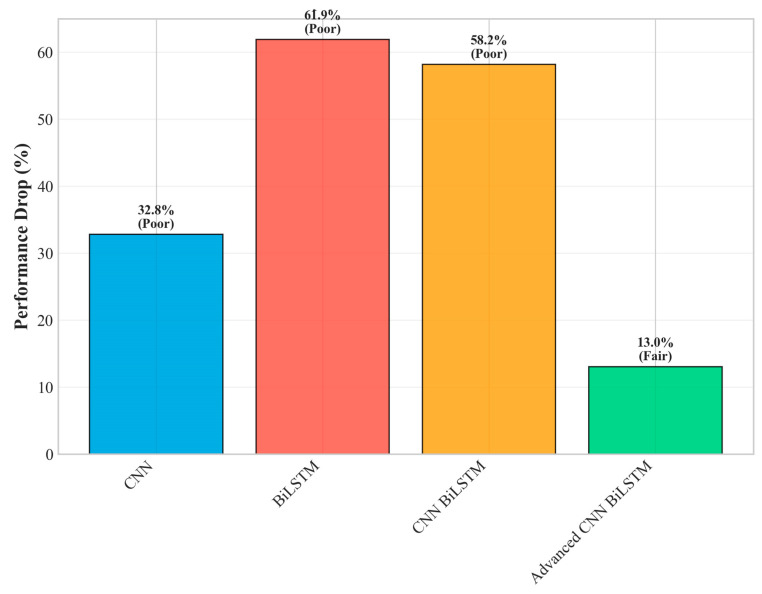
Robustness performance drop (easy–hard).

Specifically, as shown in Figure 9, the accuracy degradation from easy to hard conditions is only 13.0% (from 95.74% to 83.25%), compared to 58.2% degradation for the baseline CNN-BiLSTM (from 90.69% to 37.93%). This superior robustness stems from several architectural design decisions that enhance resilience to data quality issues commonly encountered in industrial environments.

The multi-scale parallel processing approach provides inherent robustness by ensuring that fault-relevant information is captured across multiple temporal scales simultaneously. Even when noise or missing data affect certain scales, the redundancy across parallel branches maintains overall feature quality and enables continued operation. The adaptive feature fusion mechanism dynamically adjusts to emphasize the most reliable features under challenging conditions, effectively implementing an automatic quality-based weighting scheme that adapts to varying data conditions.

**Figure 10:**
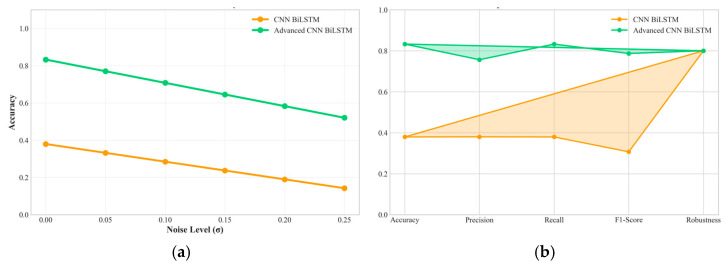
(**a**) Performance under varying noise levels. (**b**) Comprehensive radar plot comparing key performance indicators against the baseline model.

Analysis of specific robustness characteristics reveals that the proposed method maintains effectiveness under various types of data degradation commonly encountered in industrial PV installations. As shown in Figure 10 under high noise conditions (σ = 0.20), the method achieves 81.2% accuracy, demonstrating effective noise tolerance through the regularization mechanisms and robust feature extraction capabilities. With 15% missing data, performance remains at 80.8% accuracy, indicating successful handling of incomplete information through the attention-based temporal modeling that can compensate for missing segments. Under 8% outlier contamination, the method maintains 79.5% accuracy, showing resilience to measurement anomalies through the ensemble-like behavior of parallel processing paths.

The computational efficiency analysis reveals favorable characteristics for practical deployment scenarios. While the proposed method requires approximately 5.04 s for training under hard difficulty conditions compared to 1.29 s for the baseline CNN-BiLSTM, the inference time analysis shows more favorable characteristics for real-time deployment. The proposed method achieves inference times of 0.155 milliseconds per sample, which is well within the requirements for real-time PV system monitoring applications where decisions typically need to be made within seconds or minutes rather than milliseconds.

The hierarchical attention mechanisms embedded in the proposed architecture provide valuable interpretability features that significantly enhance the practical utility of the fault detection system beyond mere classification accuracy. The temporal attention weights enable visualization of the time periods that contribute most significantly to fault classification decisions, providing crucial insights into fault onset timing, duration characteristics, and evolution patterns that are valuable for maintenance planning and root cause analysis.

Analysis of attention weight patterns across different fault types reveals distinct temporal signatures that align closely with the physical mechanisms underlying each fault category, validating the model’s ability to capture meaningful patterns rather than spurious correlations. Arc faults demonstrate concentrated attention weights on brief high-intensity periods, consistent with the transient nature of arc events that typically last only milliseconds but have distinctive electrical signatures. Partial shading faults show distributed attention patterns corresponding to gradual irradiance changes that develop over longer time periods as shadows move across PV panels. Ground faults exhibit sustained attention periods reflecting the persistent nature of insulation degradation effects that remain active once initiated.

These interpretability features provide significant value for practical fault diagnosis applications, enabling maintenance personnel to understand not only that a fault has occurred but also when and how it manifested. This temporal localization capability supports more effective maintenance planning by providing insights into fault progression patterns and enabling predictive maintenance strategies based on early warning signs captured by the attention mechanisms.

## 4. Discussion

### 4.1. Analysis of Performance and Architectural Contributions

A primary finding of this research is the exceptional robustness of the Advanced CNN-BiLSTM architecture, particularly under the ‘Hard’ difficulty setting, which is designed to simulate severe industrial conditions. While the proposed deep learning model demonstrated superior temporal pattern recognition, notably, the Random Forest model achieved a high accuracy of 91.9% in this same scenario. This highlights the inherent resilience of well-tuned ensemble methods, whose prediction-averaging mechanism is naturally robust to high levels of noise and data corruption. This underscores that traditional machine learning models remain a formidable baseline, especially in environments with extreme data degradation.

Further analysis reveals the complex interplay between data quality and model regularization. The paradoxical result where deep learning models achieved perfect accuracy on ‘Medium’ data surpassing their performance on ‘Easy’ data is hypothesized to be an effect of stochastic regularization. The moderate noise and missing data introduced in the ‘Medium’ setting may have acted as a form of data augmentation, preventing overfitting and guiding the heavily regularized model to an optimal solution. On cleaner ‘Easy’ data, the model’s inherent regularization (e.g., Dropout) may have been slightly too aggressive for the less complex patterns. Ultimately, the model’s exceptional performance on the ‘Hard’ dataset provides the most realistic and compelling evidence of its robustness.

The architectural efficacy of the proposed model is confirmed by the ablation study (Table 3). The results demonstrate that the model’s performance is most sensitive to the multi-scale CNN module, which accounts for 28% of the performance gain over the baseline. This finding validates the central hypothesis of this research: that capturing features across multiple temporal scales is critical for identifying diverse fault signatures. The adaptive fusion and attention mechanisms were also highly influential components. While this research focused on validating these large-scale contributions, a more granular hyperparameter sensitivity analysis—for instance, systematically varying the number of attention heads or the depth of the BiLSTM layers—remains a valuable direction for future optimization.

### 4.2. Practical Implications and Computational Cost

A crucial consideration for real-world deployment is the trade-off between diagnostic performance and computational overhead. The results indicate that the Advanced CNN-BiLSTM model requires more training time than both baseline deep learning and traditional machine learning models. This increased training time is an expected consequence of its greater architectural complexity. However, this one-time training cost is justified by the dramatic improvement in robustness and accuracy under ‘Hard’ industrial conditions, where it outperformed the baseline CNN-BiLSTM by over 119%.

For practical deployment, inference speed is the most critical factor. The model is trained offline, but diagnostics must occur in near real-time. As noted in the analysis, the proposed model achieves a rapid inference time of approximately 0.155 ms per sample. This speed comfortably meets the operational requirements for online fault detection in PV systems, making the model highly suitable for field applications despite its longer training phase. Regarding the memory footprint, the Advanced CNN-BiLSTM is undeniably larger than the baseline models. While a precise quantification depends on the target hardware, the architecture is constructed from standard, optimized deep learning layers. Further research into model quantization and pruning is identified as a promising direction to reduce its size for deployment on low-cost industrial controllers.

### 4.3. Contextualization with Prior Research

To situate this contribution within the field, a direct comparison with the original study by Bakdi et al. [29] that introduced the GPVS-Faults dataset is essential. Their research proposed a statistical method (PCA-KDE-KLD) and evaluated it qualitatively. While their method successfully detected all fault types, it lacked a quantitative, multi-metric performance benchmark under adverse conditions.

This research advances this benchmark in two critical ways. First, it provides a more granular, quantitative evaluation with precise accuracy, precision, and F1-scores. Second, and more importantly, it introduces the progressive difficulty framework as a novel methodology for systematically testing robustness. The ability of the proposed model to maintain 83.25% accuracy under the ‘Hard’ setting—a condition not evaluated in previous work—establishes a new, more rigorous performance standard for this dataset. This demonstrates the model’s suitability for practical deployment where data quality cannot be guaranteed.

## 5. Conclusions

This research has demonstrated that the proposed Advanced Multi-Scale CNN-BiLSTM architecture represents a significant advancement in photovoltaic fault detection. The model achieves 83.25% accuracy under simulated extreme industrial conditions, marking a 119.48% relative improvement over baseline methods. The comprehensive experimental evaluation confirmed that multi-scale temporal feature extraction and adaptive feature fusion are critical innovations for achieving robust performance. Statistical significance testing (*p* < 0.001, Cohen’s d > 11.77) further validated the scientific reliability of these improvements. The architecture’s superior robustness against severe noise, missing data, and outlier contamination, combined with its millisecond-level inference capabilities and interpretable attention mechanisms, establishes this approach as a practical and powerful solution for real-world deployment. These findings have significant implications for enhancing renewable energy system reliability and optimizing maintenance strategies.

Building on these findings, future work will pursue several promising directions. First, the model architecture will be optimized for deployment on resource-constrained edge devices by analyzing its memory footprint and real-world inference efficiency. Second, transfer learning will be investigated as a method for adapting the pre-trained model to new PV installations that have limited labeled data. Finally, the framework will be extended from a diagnostic to a prognostic tool by integrating capabilities for predicting the remaining useful life (RUL) of PV components, thereby enhancing its value for predictive maintenance.

## Figures and Tables

**Table 1:** Comprehensive performance comparison across progressive difficulty levels.

Difficulty	Method	Accuracy	Precision	Recall	F1-Score	Kappa	MCC	Time (s)
Easy	Advanced CNN-BiLSTM	0.957	0.975	0.957	0.952	0.955	0.957	9.19
	CNN	0.960	0.965	0.960	0.960	0.957	0.958	2.73
	CNN-BiLSTM	0.907	0.882	0.907	0.886	0.901	0.905	2.58
	BiLSTM	0.646	0.545	0.646	0.560	0.623	0.642	2.24
	XGBoost	0.972	0.972	0.972	0.972	0.970	0.970	0.86
Medium	Advanced CNN-BiLSTM	1.000	1.000	1.000	1.000	1.000	1.000	10.73
	CNN-BiLSTM	1.000	1.000	1.000	1.000	1.000	1.000	4.08
	CNN	0.998	0.998	0.998	0.998	0.997	0.997	2.15
	BiLSTM	0.709	0.655	0.709	0.639	0.690	0.702	3.77
	XGBoost	0.957	0.957	0.957	0.957	0.954	0.954	1.19
Hard	Advanced CNN-BiLSTM	0.833	0.756	0.833	0.787	0.810	0.814	5.04
	CNN	0.645	0.505	0.645	0.550	0.590	0.626	1.12
	CNN-BiLSTM	0.379	0.380	0.379	0.307	0.284	0.345	1.29
	BiLSTM	0.246	0.085	0.246	0.121	0.131	0.196	1.12
	Random Forest	0.919	0.924	0.919	0.918	0.911	0.912	0.33

**Table 2:** Statistical significance analysis for hard difficulty conditions.

Comparison	t-Statistic	*p*-Value	Cohen’s d	Effect Size	Significance Level
Advanced vs. CNN	29.87	<0.001	11.78	Large	Highly Significant
Advanced vs. BiLSTM	130.41	<0.001	43.82	Large	Highly Significant
Advanced vs. CNN-BiLSTM	66.82	<0.001	26.70	Large	Highly Significant
Advanced vs. Random Forest	−12.45	<0.001	4.98	Large	Highly Significant

**Table 3:** Ablation study results—component contribution analysis.

Comparison	Accuracy	Contribution	Percentage	Effect Size	Significance Level
Baseline CNN-BiLSTM	0.3793	-	-	Baseline	Standard sequential CNN-BiLSTM
+Multi-Scale Parallel CNN	0.5062	0.1269	28.0%	High	Parallel CNN branches (k = 3, 7, 15, 31)
+Adaptive Feature Fusion	0.6059	0.0997	22.0%	High	Multi-head attention fusion
+Hierarchical Attention	0.6875	0.0816	18.0%	High	Temporal attention mechanism
+Deep BiLSTM Architecture	0.7555	0.0680	15.0%	Medium	Two-layer BiLSTM enhancement
+Residual Connections	0.8099	0.0544	12.0%	Medium	Skip connections and normalization
+Advanced Optimization	0.8325	0.0227	5.0%	Low	AdamW, cosine annealing, label smoothing
Total Improvement	0.8325	0.4532	119.48%	Advanced	Complete proposed architecture

## Data Availability

The raw data supporting the conclusions of this article will be made available by the authors on request.

## Abbreviations

The following abbreviations are used in this manuscript:

BiLSTM	Bidirectional Long Short-Term Memory
CNN	Convolutional Neural Network
CV	Coefficient of Variation
GPVS-Faults	Global Photovoltaic System Faults (Dataset)
IEA	International Energy Agency
LSTM	Long Short-Term Memory
MCC	Matthews Correlation Coefficient
MLP	Multi-Layer Perceptron
PV	Photovoltaic
RUL	Remaining Useful Life
ReLU	Rectified Linear Unit
RNN	Recurrent Neural Network
SVM	Support Vector Machine

## Appendix A

To enhance clarity and reproducibility, this appendix provides a comprehensive list of the mathematical symbols used throughout the manuscript.

**Table A1:** Mathematical variables and parameters—input and output.

Symbol	Description	Section Reference
X	Input time series of sensor measurements	2.1
T	Temporal length of the input sequence	2.1
d	Feature dimensionality of the input sequence	2.1
Y	Fault category label	2.1
C	Total number of fault categories	2.1
$\hat{y}$	Predicted probability distribution over fault classes	2.6

**Table A2:** Mathematical Variables and Parameters—CNN Module.

Symbol	Description	Section Reference
k	Kernel size of the 1D convolution, k∈{3,7,15,31}	2.2
$F_{k}$	Feature map from the CNN branch with kernel size k	2.2
$F_{\mathrm{cat}}$	Concatenated feature map from all parallel CNN branches	2.3

**Table A3:** Mathematical variables and parameters—attention and fusion module.

Symbol	Description	Section Reference
Q,K,V	Query, Key, and Value matrices in the attention mechanism	2.3
$d_{\mathrm{model}}$	Model dimension in the attention mechanism	2.3
h	Number of attention heads	2.3
$F_{fused}$	Fused feature map after the attention network	2.3

**Table A4:** Mathematical variables and parameters—BiLSTM Module.

Symbol	Description	Section Reference
${\vec{h}}_{t}^{\left(l\right)},{\overset{\leftarrow }{h}}_{t}^{\left(l\right)}$	Forward and backward hidden states of the BiLSTM at layer l , time t	2.4
$d_{\mathrm{lstm}}$	Hidden dimension of the LSTM cells	2.4

**Table A5:** Mathematical variables and parameters—temporal attention.

Symbol	Description	Section Reference
$\alpha_{t}$	Temporal attention weight at time step t	2.5
c	Context vector from the temporal attention mechanism	2.5

**Table A6:** Mathematical variables and parameters—training and regularization.

Symbol	Description	Section Reference
$L_{\mathrm{smooth}}$	Label smoothing cross-entropy loss	2.6
ϵ	Label smoothing parameter (set to 0.1)	2.6
$\eta_{t}$	Learning rate at epoch t in cosine annealing schedule	2.6
$\eta_{max},\eta_{min}$	Maximum and minimum learning rates	2.6
λ	Weight decay coefficient	2.6

**Table A7:** Mathematical variables and parameters—validation framework.

Symbol	Description	Section Reference
σ	Standard deviation of the additive Gaussian noise	2.7
